# Supplementary material for: Racial & ethnic disparities in geographic access to critical care in the United States: A geographic information systems analysis
Source: PLoS One. 2023 Nov 1;18(11):e0287720. doi: 10.1371/journal.pone.0287720 (PMC10619775; doi:10.1371/journal.pone.0287720)
Supplement: S1 File — (DOCX) [file pone.0287720.s001.docx]

**Racial & Ethnic Disparities in Geographic Access to Critical Care in the United States**

Online Data Supplement

**Supplemental Figure 1.** Diagram displaying block group centroid, ICU Catchment Area, and ICU ratio calculation^.^ For block group centroids located within more than one ICU catchment area, the block group ratio reflects the average population served by the various ICUs from which they can receive care


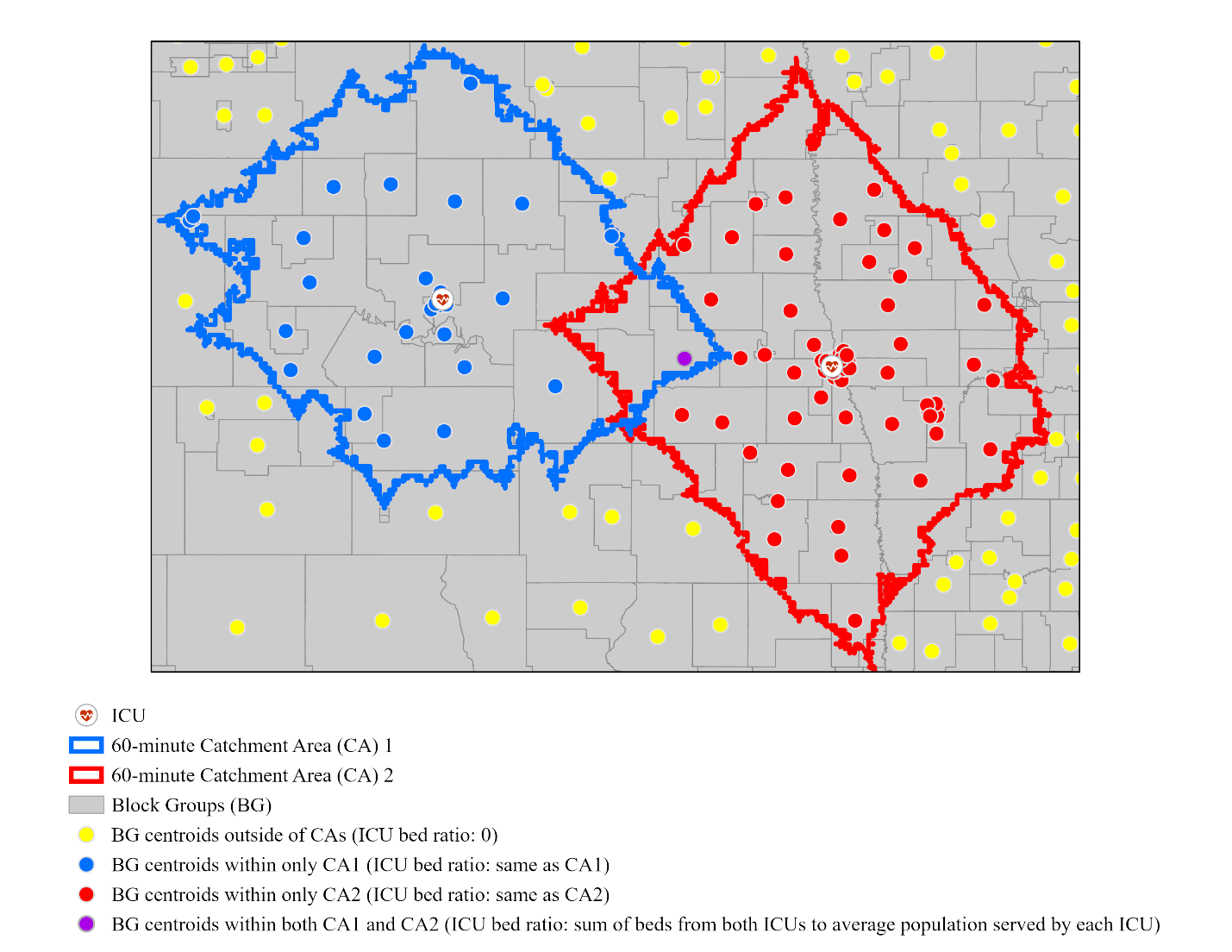


**Supplemental Table 1A**. Population counts for 30-minute drive time

**Supplemental Table 1B**. Population counts for 90-minute drive time

**Supplemental Figure 2A.** ICU beds per 100,000 adults available at the US Census block group level within 30-minute drive time


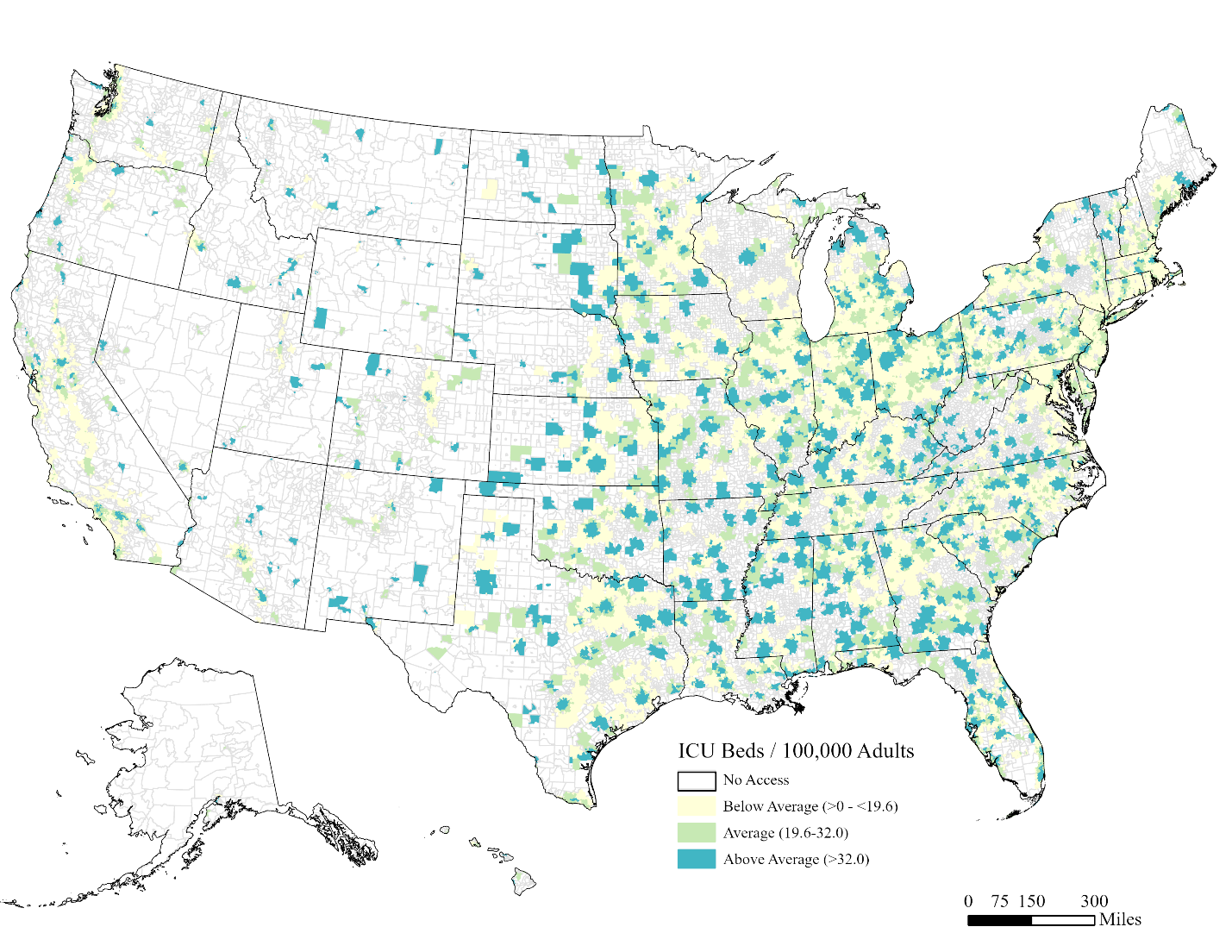


**Supplemental Figure 2B.** ICU beds per 100,000 adults available at the US Census block group level within 90-minute drive time


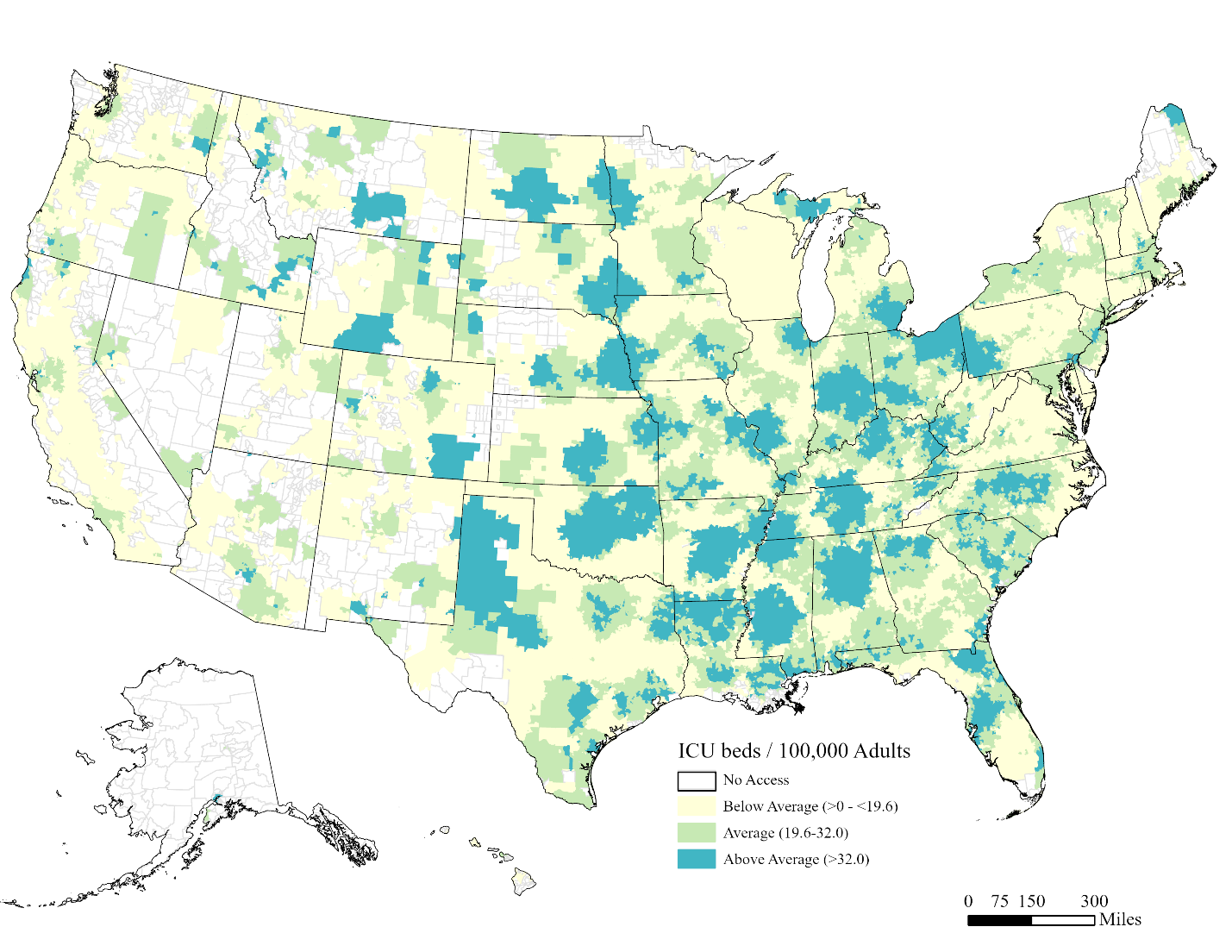


**Supplemental Figure 3A.** No access and below average ICU bed access based on 60-minute drive time at the state level and by race/ethnicity

**Supplemental Figure 3B.** Average and above average ICU bed access based on 60-minute drive time at the state level and by race/ethnicity
